# Spectroscopic investigations to reveal synergy between polystyrene waste and paraffin wax in super-hydrophobic sand

**DOI:** 10.1038/s41598-023-36987-4

**Published:** 2023-06-17

**Authors:** K. Al-Mokhalelati, F. Karabet, A. W. Allaf, M. Naddaf, A. G. Al Lafi

**Affiliations:** 1 Department of Chemistry, Faculty of Science, Damascus University, Damascus, Syrian Arab Republic; 2 Department of Chemistry, Atomic Energy Commission, P.O. Box 6091, Damascus, Syrian Arab Republic

**Keywords:** Environmental chemistry, Environmental sciences, Chemistry, Materials science

## Abstract

Sand based superhydrophobic materials, such as paraffin-coated sand, are the focus of global research to fight land desertification. The present work investigates the development of paraffin-coated sand with extending service life as well as improving and stabilizing hydrophobic property by adding plastic waste. While the addition of polyethylene (PE) did not improve the hydrophobic property of paraffin coated sand, incorporating 4.5% of polystyrene (PS) in the composition of coated sand increased the contact angle. Fourier Transform Infrared spectroscopy (FTIR), X-ray diffraction patterns (XRD) and two-dimensional correlation spectroscopy (2D-COS) indicated that PS increased the molecular orientation of sand and reduced the thickness of the paraffin coating. Paraffin on the other hand improved the distribution of PS and prevented aggregation with sand. Both FTIR bands at 1085 cm^−1^ and 462 cm^−1^ were more sensitive to change in PS contents, while other bands at 780 cm^−1^ and 798 cm^−1^ were more sensitive to change in paraffin contents. Moreover, the XRD patterns of sand were split into two components by the addition of PS indicating the transformation of morphology to less ordered or more distorted form. 2D-COS is a powerful tool to reveal harmony of components in mixtures, extract information related to the role of each of them, and help in decision-making process regarding choosing the appropriate recipes.

## Introduction

Land desertification is among the most serious environmental issues challenging the world economy and all living species on the Earth^1^. In a low humidity environment such as the desert, the water in air is very limited, and the soil is rigorously waterlogged. Even on rainy days, the sand absorbs rainwater massively but it evaporates without any traces in the sunshine due to high temperature as well as due to the fact that sand is naturally hydrophilic material^2^. Global efforts have been directed to produce cheap and readily available hydrophobic sand based materials, that are capable of preventing water leakage and at the same time ensure air permeability to promote the root aeration of plants^3^. Therefore, varieties of fabricated methods and low water affinity materials have been utilized. Artificial plastic mulches have been applied with limited success due to their high cost, non-biodegradability and their possible contribution to micro plastics pollution issues^4,5^. Atta et al., have obtained excellent water repellency using paraffin wax capsules comprising hydrophobic silica nanoparticles for coating desert sand. They have achieved a great water-holding capacity by mixing of the superhydrophobic sand with untreated sand^6^. Liu et al.^7^ have produced a superhydrophobic sand particle through coating the sand particles with photocatalytic nanoparticles and a self-assembled monolayer of hydrophobic material. They found that superhydrophobic sand could result in a significant reduction of the evaporation loss of water, which is very promising preservation and purification of water in real applications. Moreover, Liu et al., fabricated super-hydrophobic sand having water contact angles between 153° and 163° following two procedures; polymerization of dopamine hydrochloride^8^ and bonding SiO_2_ nanoparticle onto the sand surface^9^. In addition, Mosayebi et al.^10^ fabricated super-hydrophobic sand with water contact angle of 152° by chemical vapor deposition of polydimethylsiloxane (PDMS) on the surface of sand. Purified paraffin waxes offer biodegradable, nontoxic, and inexpensive coating materials at an industrial scale^11^. Gallo et al.^12^ applied dip coating technique to coat arid region sand with paraffin wax for water-use efficiency and agricultural applications. However, these coatings suffered from short service life, considered mechanically unstable and they did not adhere well to the sand surface^6^.

On the other hand, recycling of commodity plastic wastes such as polyethylene (PE), polypropylene (PP) and polystyrene (PS) rather than landfill them prevents leakage of toxic substances into the soil, and subsequently benefits both the local and extended ecosystem^13,14^. Various attempts including material, thermal and chemical recycling^15–17^ have been verified to deal with the disposal of already littered plastic wastes. One merit of the present work was to investigate the effects of adding plastic wastes to the composition of paraffin-coated sand. The plastic wastes may play a role in increasing the service life of the coated sand, which has been reported recently^12^. In addition, the plastic wastes may contribute to the hydrophobic nature of paraffin-coated sand due to their non-polar chemical structure and amorphous morphologies.

It is of prime importance to understand the mechanism by which the sand become hydrophobic and the structural changes associated with that. Thus, a spectroscopic study is beneficial to reveal molecular level alterations. The use of two-dimensional correlation spectroscopy (2D-COS) has advantageous to the present work as it helps in the separation of the overlapped bands and assigning them to the corresponding component. In addition, 2D-COS helps in decision-making process regarding choosing the appropriate component to be added. Yet, obtaining information on a molecular level and correlating the data from different analytical probes are other merits of 2D-COS^18–21^.

In this work, sand was coated with paraffin wax and polymers, namely PS and PE. According to our knowledge, the procedures for preparing and characterizing hydrophobic materials starting from Syrian arid sand have not been reported before. Five different coating systems were investigated; these are paraffin alone, PS alone, PE alone, paraffin with PS, and paraffin with PE. The samples were characterized by Fourier Transform Infrared, X-ray diffraction, contact angles measurements, and scanning electron microscopy. Analysis of the data was carried out by mean of two-dimensional correlation spectroscopy to correlate between hydrophobicity and structure/morphology of the coated sand.

## Experimental

### Materials and coating of sand

Sand samples were collected from Al-Qaryatayn area near Homs city on the outskirts of the Syrian Desert. The sand was washed with tap water, filtered and then dried overnight in an over at a temperature of 50–65 °C. Finally, the sand was sieved using the sieving machine. Paraffin wax (melting point 58–60 °C, C_n_H_2n+2_, where, n = 20–30, Avonchem) was used as a coating agent. Polystyrene (PS) and polyethylene (PE) wastes, which are usually used in food packaging (Styrofoam) were collected from local area and used as second coating agents. N-hexane (boiling point (b.pt.) 69.0 °C), dichloromethane (b.pt. 39.6 °C) and toluene (b.pt. 110.6 °C) were used as solvents for paraffin, PS and PE respectively. They were obtained from Sigma-Aldrich, Germany, and used without further purification. Surface coating experiments were carried out by shaking the flasks (containing 10 g of sand added to 15 mL of n-hexane) at 150 rpm for predetermined time using magnetic shaker. Five coating systems were investigated, as quoted in Table 1. Each system contained five coated sand samples.

**Table 1:** Samples code and composition of coating.

Samples designation	Composition (w%)		
	Paraffin wax	PS	PE
System (I)	4.5–19.2 (0.5–2.5 g)	4.5–3.9 (0.5 g)	0
System (II)	4.5–3.9 (0.5 g)	4.5–19.2 (0.5–2.5 g)	0
System (III)	4.5–19.2 (0.5–2.5 g)	0	4.5 (0.5 g)
System (IV)	4.8–16.7 (0.5–2.0 g)	0	0
system (V)	0	4.8–16.7 (0.5–2.0 g)	0

Weight of sand was fixed at 10 g in all systems.

It should be pointed out here that at least three replicates were carried out for each prepared sample.

### Characterizations

Structural alterations were investigated by FTIR spectroscopy (Thermo Scientific Nicolet 6700 FT-IR Spectrometer, Madison, USA) operated with a DTGS detector. The KBr method was used and the spectra were acquired from 400 to 4000 cm^−1^ at a resolution of 2 cm^−1^ and 200 scans. A separate background spectrum was subtracted in each data collection.

Crystallinity and morphology of the sand samples were investigated using powder X-ray diffraction (XRD), and scanning electron microscopy (SEM) respectively. A transmission diffractometer (STADI-P STOE, Darmstadt, Germany) equipped with CuK_α_ radiation ($\lambda = 1.54060$ Å) and a germanium monochromator was utilized. The machine was operated at 50 kV and 30 mA, and XRD patterns were recorded from 2θ = 10° to 2θ = 90° with a scanning step of 0.02°. A scanning electron microscope (Tescan Vega II XMU, USA) operated at 20 kV was utilized to capture the surface morphology of sand particles.

The sessile drop method was used to measure the contact angles utilizing an OCA 15 plus, SCA 20 (Data Physics Instrument GmbH, Germany). The digital drop images were handled using an image analysis system that calculated both the left and right contact angles from the shape of the drop with an accuracy of ± 0.1°. A disc of each coated sand sample was prepared by pressing the sample at 1 ton, the contact angle was then measured by depositing a double distilled water droplet (3 μL) onto the resulted disc.

Each measurement was carried out for at least three replicates, and the average results are given throughout the work.

### Data pretreatments and 2D correlation analysis

To improve the quality of the 2D-COS spectra, all FTIR spectra were baseline corrected using OMNIC software package, smoothed with a Savitzky–Golay smoothing function of two polynomials and 15 points, and finally normalized based on a reference band^21–23^. The band at 694 cm^−1^ has been attributed to Si–O of SiO_4_ symmetrical bending, and its presence confirms the crystalline nature of quartz^24,25^. This band was present in all sand samples and was not affected by the additives, thus it was chosen as a reference band. Similar data pretreatments were carried out on the XRD patterns taking the peak at 26.6° as a reference^26^. The data were arranged to obtain FTIR and XRD data sets in which one perturbation was considered. The dynamic spectra of each data set were obtained by subtracting a reference spectrum taken as the spectrum of the sample with maximum perturbation, i.e. maximum contents of PS or paraffin. The calculation and visualization of the 2D spectra (synchronous and asynchronous) were achieved with the help of the 2DShige version 1.3 software (Shigeaki Morita, Kwansei-Gakuin University, 2004–2005) based on a discrete Hilbert transform algorithm developed by Noda^27–29^. The 2D-COS spectra consist of positive (shown in white/red areas) and negative (shown in gray/blue areas) cross-peaks in both synchronous and asynchronous spectra, and they were interpreted by means of Noda’s rule^29^.

## Results and discussion

### Wettability of coated sand

**Figure 1:**
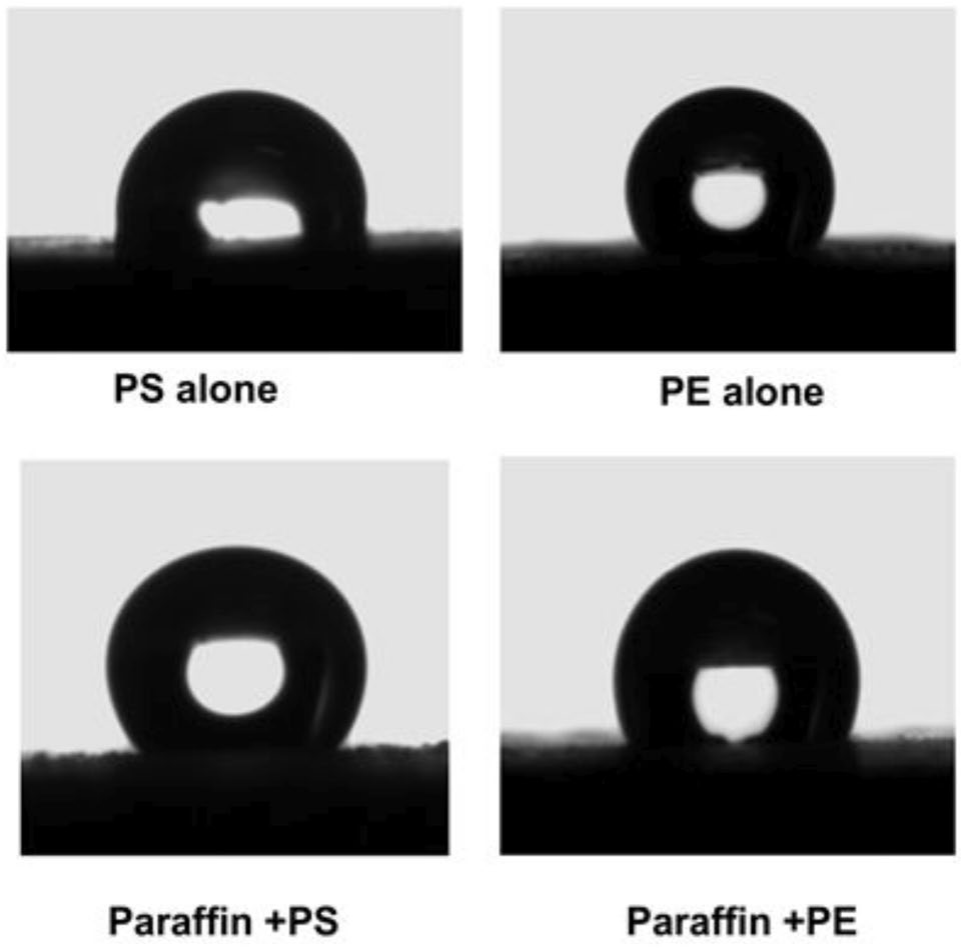
Optical photos of a water droplet sitting on a horizontally leveled surface of the hydrophobized sand by different materials as indicated.

All samples from different systems were first screened for wetting properties by carrying out the contact angle measurements. Figure 1 shows the images of water droplet as it was placed onto the surface of different compositional hydrophobized sand discs. The use of PE coating alone showed better hydrophobicity than the PS one. However, the addition of the paraffin to PS substantially improved the hydrophobicity of the sand, while its addition to PE did not show considerable effect.

**Figure 2:**
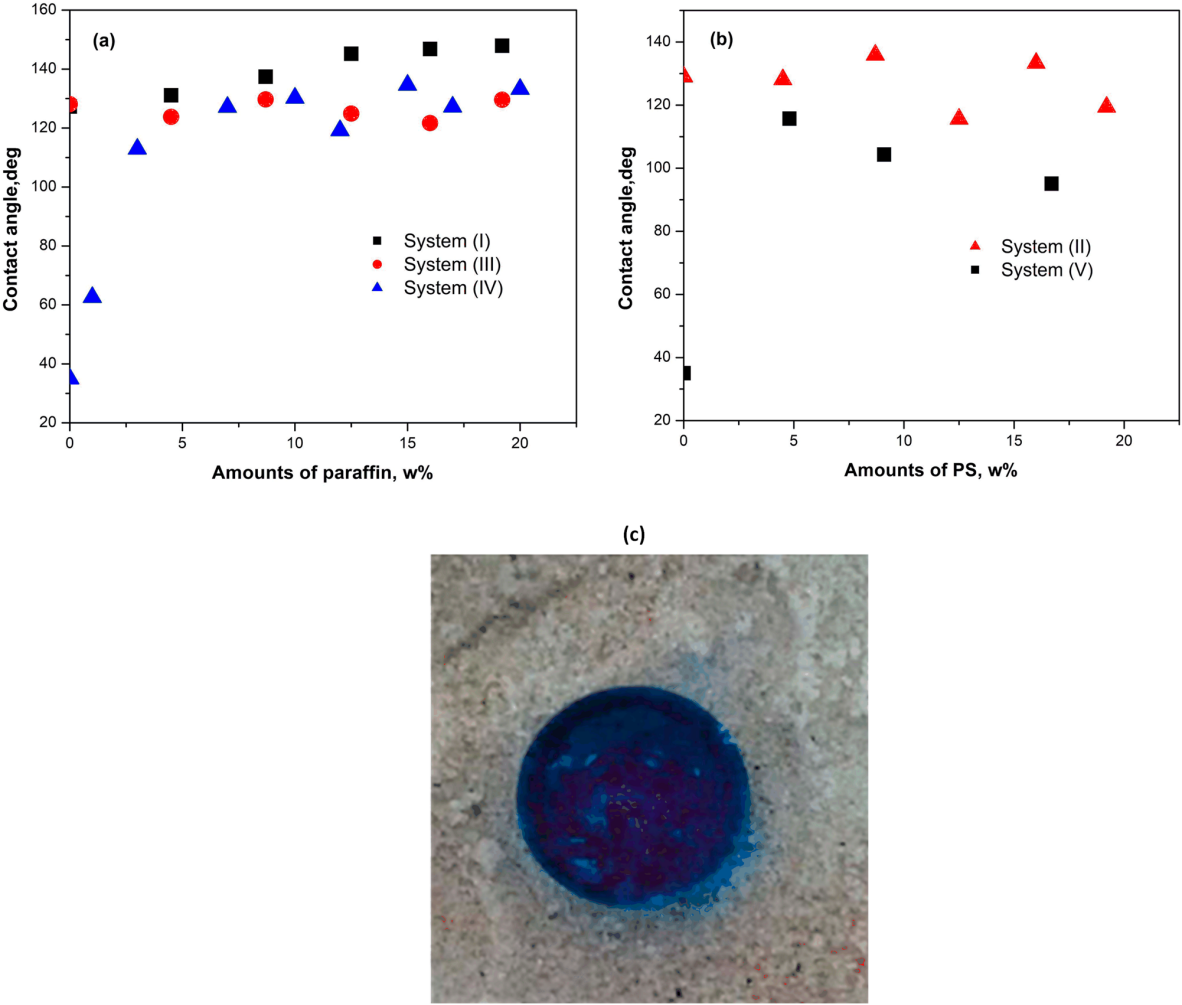
The contact angle values against the composition of coated sand: (**a**) effects of paraffin, (**b**) effects of PS, and (**c**) an optical graph of a blue ink colored water droplet on the surface of 4.5% PS-paraffin coated sand.

Figure 2a shows the variation of water contact angle (CA) of different coated sand samples as a function of additive concentrations (weight percentages, w%). The contact angle increased from 35° (for ordinary sand) to 125°, 128° and 122° for 4.5% PS, 4.5% PE and 4.5% paraffin coated sand respectively. After that, the contact angle decreased with the increase of PS percentage (system II), see Fig. 2b, but it became nearly constant with increasing the percentage of paraffin (system IV). It was also clear that adding increasing amount of paraffin at a constant loading of PS (system I) improved the contact angle, which reached a value of 145° when the paraffin percentage reached 19.2%. Figure 2 also reveals that there was a clear trend with added paraffin while the data were more scattered with the added PS. This could be due to phase separation in the PS coated sand and the aggregation of sand particles, which is due to the adhesion properties of PS. Thus, paraffin played a positive role in PS-coated sand by preventing aggregation and phase separation of the composite.

It was evident from Fig. 2b that increasing the content of PS decreased the contact angles, and the PS-coated sand with 4.5% paraffin (system II) and the PE-coated sand with 4.5% paraffin (system III) had lower contact angles in comparison with paraffin coated sand. At low paraffin content, i.e. 4.5% the addition of 4.5% PS improved the contact angle of the sand dramatically, but this levelled off with increasing paraffin content. However, above 12.5% paraffin loading, the sand with 4.2–3.9% PS had slightly higher contact angles than similar samples without PS. It could be argued that PS with higher free volume, higher polarity and lower ability to crystalline than PE played a role in air bubbles stabilization and homogeniousity of paraffin coating on sand.

Since all contact angles measurements were carried out on discs of coated sand (pressed sand), it should be mentioned that examination of a sample without pressing yielded more than 10° increase in the contact angle. This was due to the role of air bubbles and voids in the structure of coated sand that were decreased by pressing the samples. The effect of pressing process is clearly clarified in Fig. 2c, which represents a photograph of a blue ink colored water droplet (50 µL) sitting on the surface of unpressed sand modified with coating system (I). The surface shows a superhydrophobic state with contant angle more than 150°. Similar superhydrophobic behavior has been shown by Liu et al.^7^ for hydrophobized sand surface coated with the functional TiO_2_ or ZnO and a self assembled monolayer of octyltrimethoxysilane (OTS). They found that the evaporation loss of water could be reduced by 60–90% after coating.

It was concluded that both paraffin and PS had an effect in producing and stabilizing the hydrophobizied sand. To understand the role of each of them, a detailed investigation was carried out considering both system (I) and system (II).

### FTIR spectroscopic study

**Figure 3:**
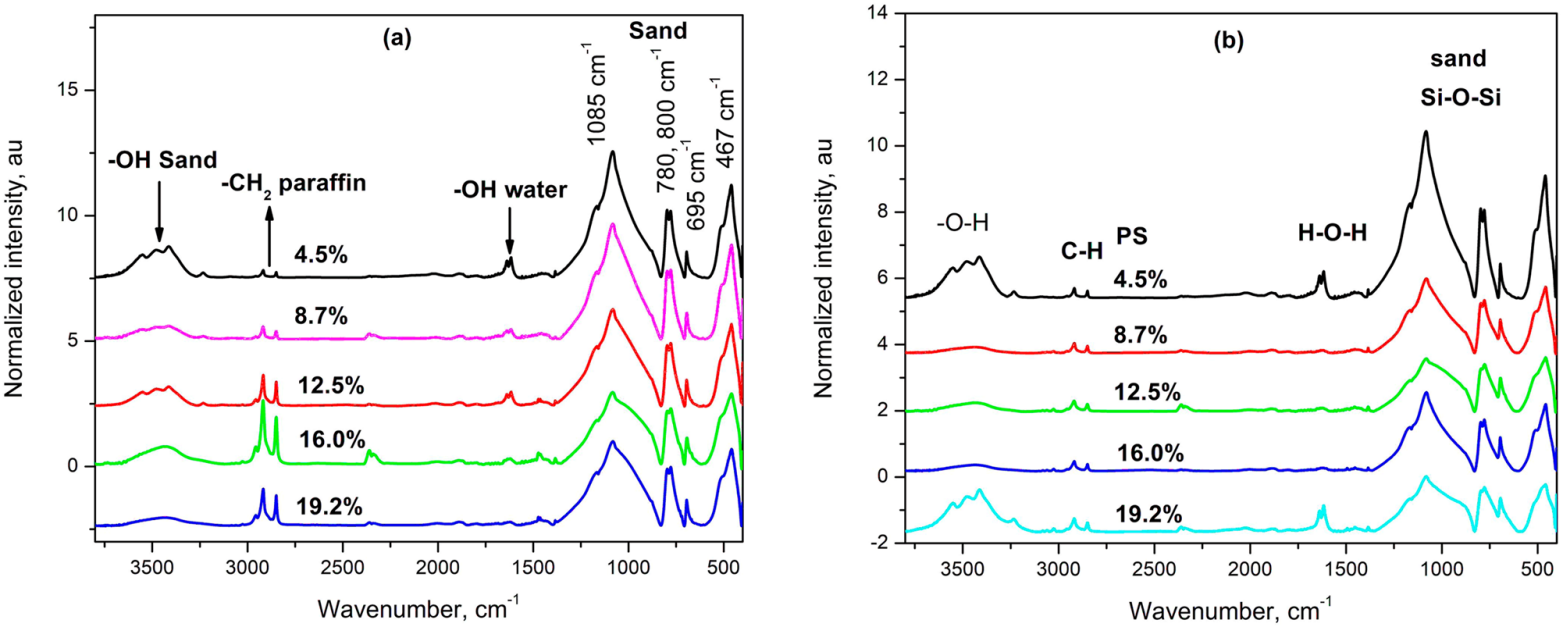
FTIR spectra of natural sand and the effects of coating with (**a**) paraffin and (**b**) polystyrene.

The FTIR spectra of paraffin and polystyrene-coated sands are shown in Fig. 3a and b respectively. The FTIR spectrum of sand comprised mainly absorption bands at 3300–3500, 1600–1640, 1085, 800, 780, 695, 514 and 467 cm^−1^. The stretching vibration of hydroxyl groups (–OH) occurs in the region from 3300 to 3500 cm^−1^. They are originated from water molecule (located at the surface and crystallized water within the structure of silica) and silanol groups (Si–OH), which are mostly located at the surface of the silica particles. The adsorption band in the region from 1600 to 1640 cm^−1^ is attributed to the bending or deformation mode of molecular coordinated water adsorbed to the Si–O–Si structure^30^. The change in the intensity of –OH stretching mode reflects alterations in the abundance of both silanol and molecular water, whereas the intensity changes of the H_2_O bending mode indicates that the amount of molecular water in the alteration layer is changed^31^. The broad band in the region from 1050 to 1100 cm^−1^, which is centered at 1085 cm^−1^ in this work, has been assigned to asymmetric stretching vibrations of Si–O–Si bonds. Broadening of this region has been attributed to lower internal order of the material, while a peak shift towards lower wavenumbers has been assigned to the formation of Si–O-Metal bonds^32^. Moreover, shifting of the bands in this region to lower wavenumbers indicated the increasing Al:Si ratio in the sample and an absorption band between 1018 and 975 cm^−1^ has been attributed to stretching vibrations of the Si–O–Al bonds^30^.

**Figure 4:**
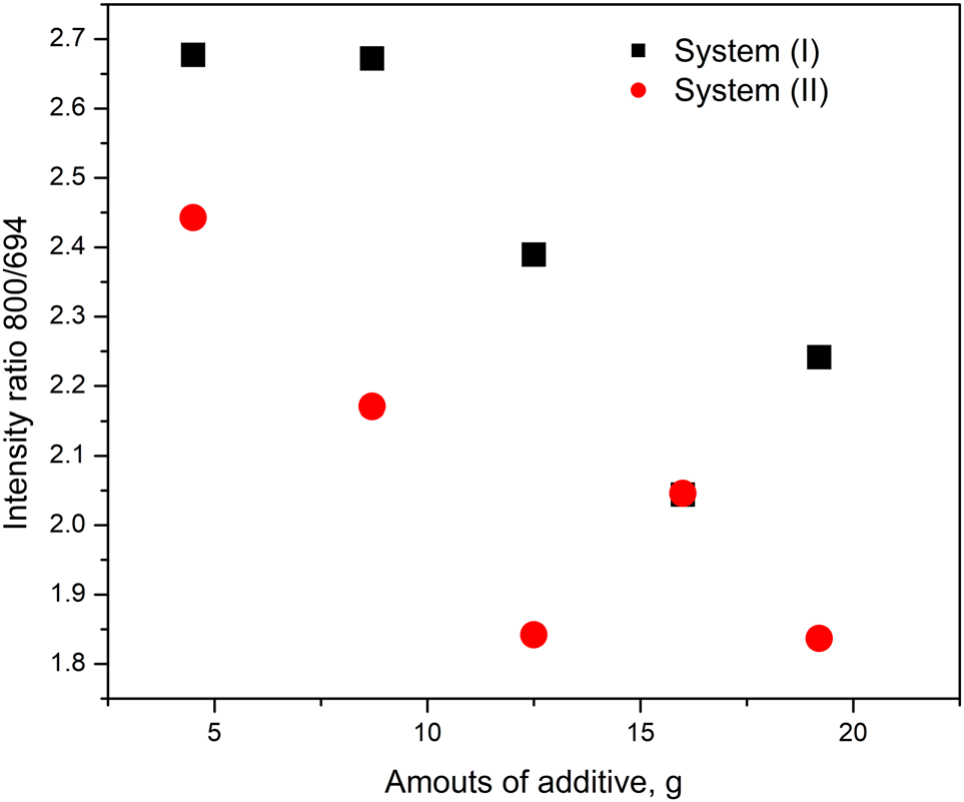
The effects of additives on the intensity ratio 800/694.

The absorption bands at 780 and 800 cm^−1^ are attributed to Si–O–Si symmetrical stretching vibration in quartz. They are particle size dependent, and indicated the presence of crystalline silica^33^. The separation of these two band is maximum with particle size less than 2 micron^34^, but their resolution decreased with increasing particle size and eventually the two bands merged at 800 cm^−1^. In addition, these two bands have been reported as the most suitable bands for quantitative analysis of silica^35^. The two bands at 467 and 514 cm^−1^ are common to many silicate minerals^33^. The band at 467 cm^−1^ is attributed to Si–O–Si out of plane deformations and that at 514 cm^−1^ is attributed to O–Si–O bending mode^30,35^. The band at 694 cm^−1^ has been attributed to Si–O of SiO_4_ symmetrical bending, and its presence confirms the crystalline nature of quartz^24,25^. The absorption ratio 800/694 has been used to evaluate the crystallinity of the quartz samples^36^. The absorption ratio 800/694 was plotted against the amounts of additives in the coated sands and was presented in Fig. 4. It was evident that both additives decreased the intensity ratio and thus increased the crystallinity of sand probably by increasing the molecular orientation. However, the effects of polystyrene were more pronounced and the increase in crystallinity with paraffin as an additive occurred only after the addition of 8.7%w.

### Morphological study

**Figure 5:**
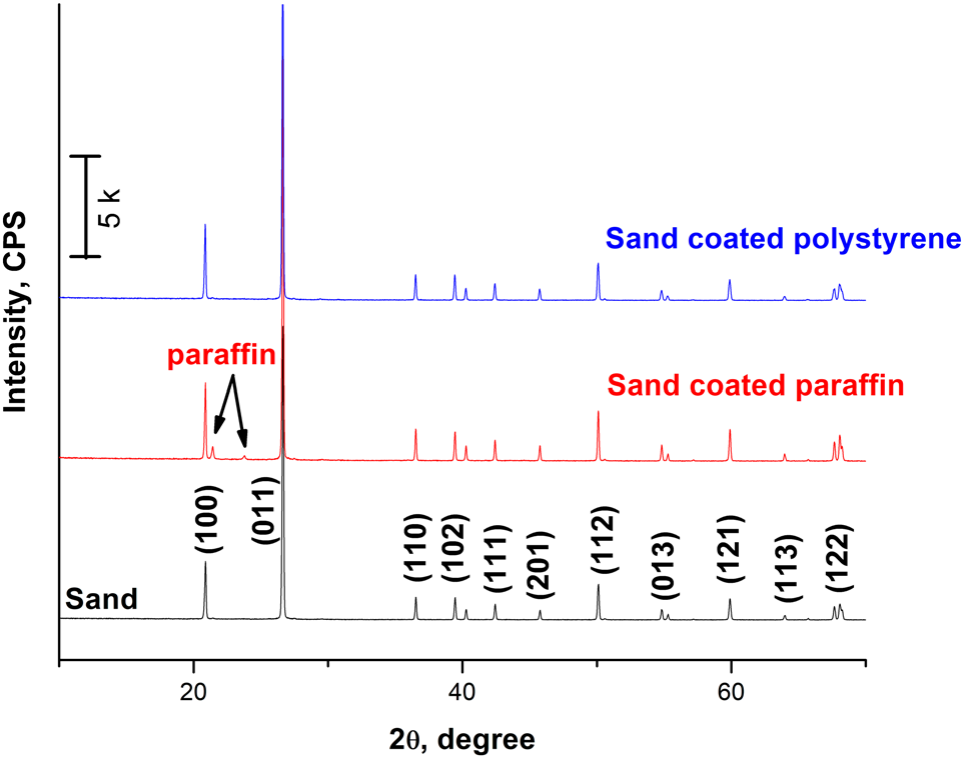
The XRD patterns of sand and coated sands.

Figure 5 shows the XRD patterns of natural silica sand as well as silica sand after being coated with paraffin wax, and poly styrene. The XRD patterns of sand are indexed to quartz^24,25,37^. Both diffraction peaks intensities and positions show no changes, which indicated that there was no chemical reaction had taken place. The two new peaks appeared at 21.42° and 23.80° that increased with increasing paraffin: sand ratio were indexed to paraffin, which has another peak at 26.6° but highly overlapped with sand peak at 26.7°^38^. No patterns could be observed for PS confirming the amorphous nature of this additive.

**Figure 6:**
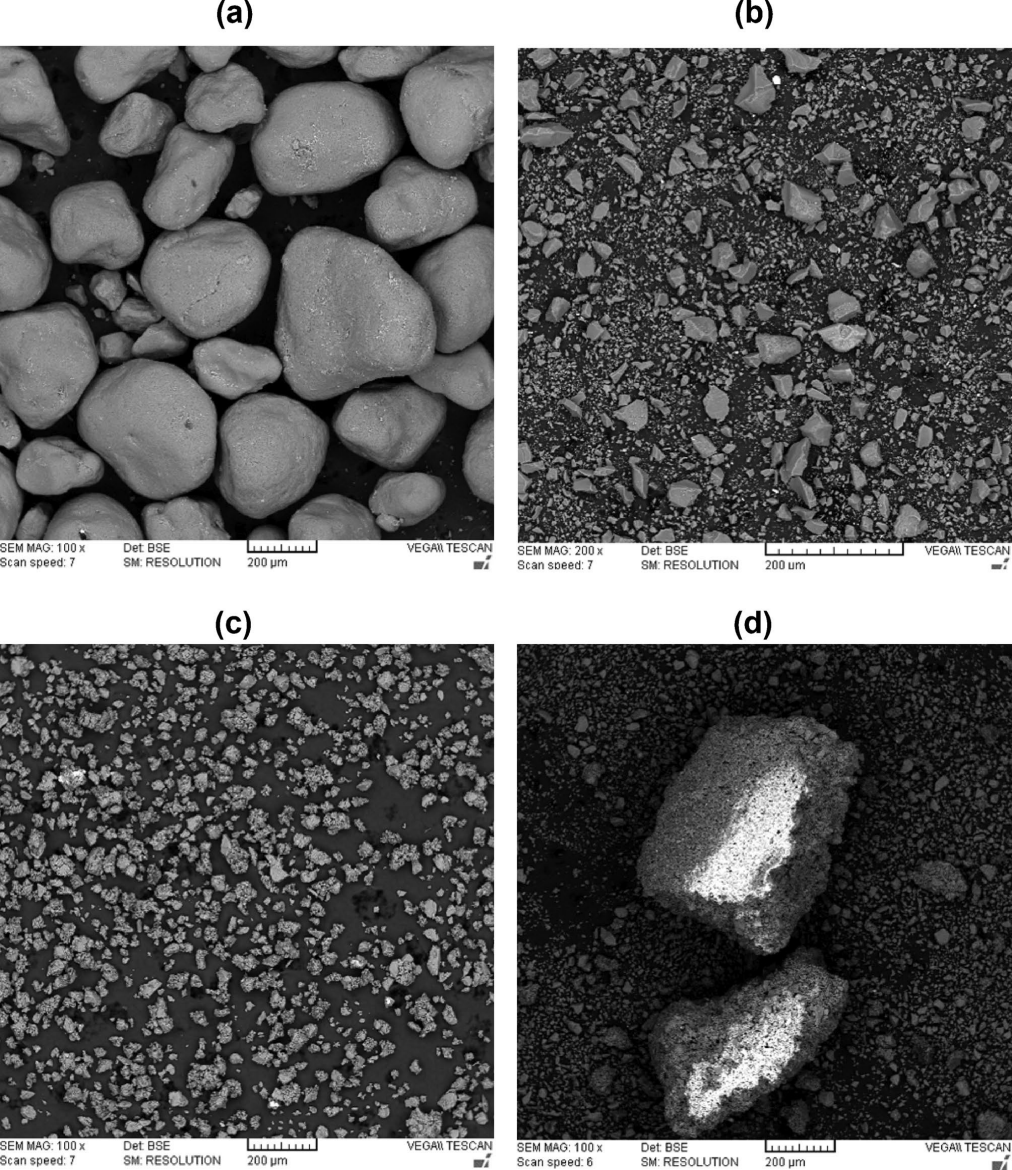
The SEM images of natural sand (**a**), granulated natural sand (**b**), paraffin coated sand (**c**) and PS coated sand (**d**).

The SEM images show the particles of natural sand, Fig. 6a, with an average diametre of 200 µm, while Fig. 6b shows the same sand particle after crushing and sieving them to the size of less than 90 µm. The diametres of crushed sand particles were in the range from 10 to 50 µm. The difference between the two additives was apparent in Fig. 6c and d, in which coating with PS was characterized by aggregation of sand particles while coating with paraffin produced smaller well separated aggregate. The consequences of such morphologies were the inhomogeniousity of the PS-coated sand which resulted in the scattered results obtained from FTIR and XRD analysis.

In summary, the hydrophopic characteristics of coated sand accompany with reduction in hydroxyl groups, crystallization of paraffin over the sand particles, ruffness of the surfaces and the apsence of coated sand particles aggregation.

### 2D-Correlation spectroscopic (2D-COS) analysis

Further indepth investigations were carried out by analyzing both FTIR and XRD data using the concept of 2D-COS. As the main purpose of the analysis was to understand the effects of PS and the role of paraffin in reducing the hydrophilicity of sand, the concepts of hybrid and hetero 2D-COS were utilized.

#### Hybrid 2D-COS

**Figure 7:**
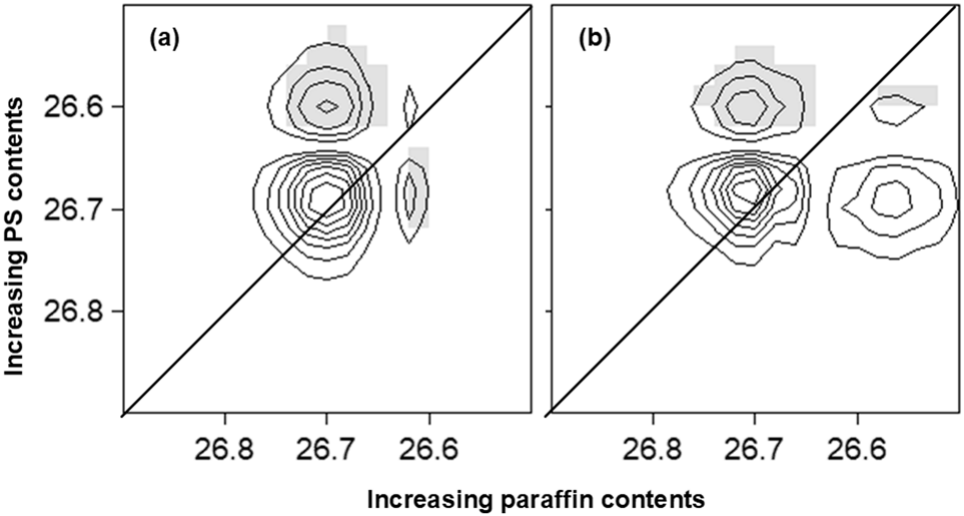
(**a**) Synchronous and (**b**) asynchronous hybrid 2D correlation maps in the range from 26.5° to 30° constructed from the intensity of XRD patterns of sand coated paraffin/PS with varying amounts of PS or paraffin.

With the prior knowledge and understanding of the mechanism of properties changes in the corresponding materials, one merit of hybrid 2D-correlation is the help in the making decision process to choose the appropriate additive. The hybrid correlations of the XRD patterns were focused on the region from 20° to 30°, in which characteristic peaks of both sand and additives (paraffin and PS) were present, see Fig. 7a and b.

In the synchronous map in Fig. 7a, four cross-peaks were observed, two of which were positive that correlated the peaks at (26.7, 26.7)° and (26.6, 26.6)° The others were negative and correlated the peaks at (26.7, 26.6)° and (26.6, 26.7)°. The peak at 26.6° was indexed to paraffin^38^, while the peak at 26.7° was indexed to sand. The positive cross peak reveals that the intensities of both peaks at 26.7° and 26.6° change in-phase, although they arise from the two different systems. Increasing paraffin contents led to the observed increase in the intensity of the peak at 26.6° and the intensity decrease of the peak at 26.7° in system (I) (increasing paraffin at constant PS content). This was confirmed by the presence of negative cross peaks at (26.7, 26.6)° and (26.6, 26.7)°. In addition, the decrease in the intensity of the peak at 26.7° in both systems indicated the decrease in the overall crystallinity of the sand. However, the positive cross peak at (26.6, 26.6)° indicated that paraffin increased in both systems but the content of the later was fixed at 4.5% and the contents of PS were varied in system (II). To explain these observations, it was suggested that the role of PS in the two systems was to reduce the crystallinity of sand and reduce the thickness of the paraffin coating, which implied more homogeneous coating. Therefore, the observed increase in the peak intensity at 26.6° in system (II) was due to the transformation of the peak at 26.7°. Moreover, applying the sequential order rule by observing the signs of correlation peaks in both synchronous and asynchronous (Fig. 7b) maps led to the following order of peak change: 26.7° (system (I)) → 26.7° (system (II)) → 26.6° (system (II)) → 26.6° (system (I)). Thus, the presence of 4.5% of PS reduced the crystallinity of sand more than the presence of 4.5% of paraffin, and the change in morphology of sand occurred to further extent with increasing PS content than increasing contents of paraffin.

Similar conclusions were obtained by analyzing the region from 20.6° to 21.6^o^ in which six cross peaks were present; three of them were positive and the others were negative. The positive cross peaks at (20.9, 20.9), (21.4, 20.8) and (20.8, 20.8)° indicated that paraffin and sand peaks at 21.4° and 20.8° respectively, increased in both systems. On the other hand, the negative peaks at (21.4, 20.9), (20.8, 20.9) and (20.9, 20.8)° indicated that the sand peak at 20.9° decreased in both systems. These were consistent with the previous region such that the addition of both paraffin and PS reduced the crystallinity of sand and PS played a role in improving the coverage of paraffin coating. Moreover, it seems that the sand peak at 20.9° shifted to 20.8° and the later increased with the increase of both additives consistent with phase transformation of sand.

**Figure 8:**
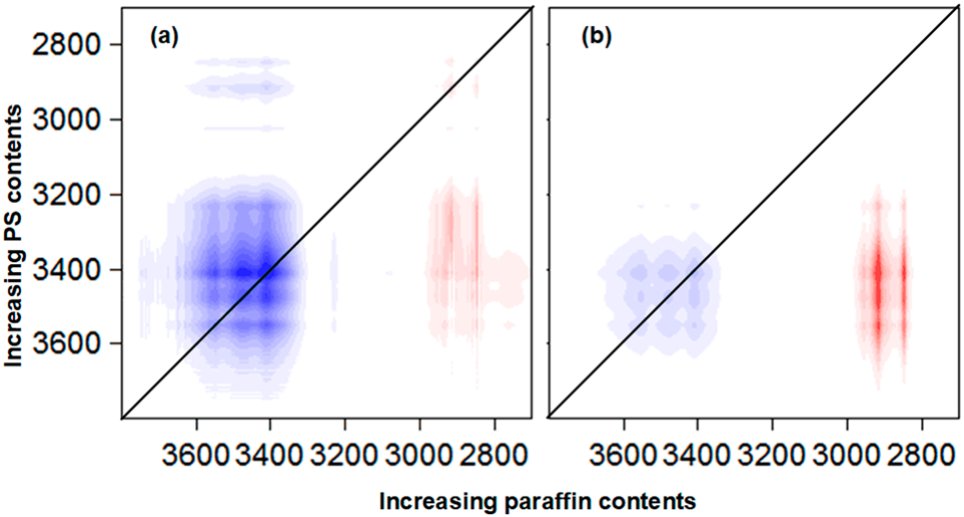
(**a**) Synchronous and (**b**) asynchronous hybrid 2D correlation spectra in the range of 3700–2700 cm^−1^ constructed from the intensity of FTIR bands of sand coated paraffin/PS with varying amounts of PS or paraffin.

The hybrid correlations of the FTIR spectra were focused on the regions from 3700 to 2700, from 1600 to1800 and from 1200 to 400 cm^−1^ where most changes that are associated with surface hydroxyl groups, additives bands and morphology of sand are expected to manifested themselves. All cross-peaks that correlated the hydroxyl groups with each others; i.e. at wavenumbers above 3200 cm^−1^ were negative, see Fig. 8a and b, indicating the opposite direction of changes of hyroxyl intensity in the two studied systems. It was shown that the intensity of hydroxyl groups decreased with increasing paraffin contents at constant loading of PS, thus the intensity of these groups increased with the addition of PS. Two interesting cross peaks were observed; one was positive that correlated the bands at (2930, 3500) cm^−1^ and the other was negative that correlated the bands at (3500, 2930) cm^−1^. The band at 2930 cm^−1^ in system (I) was attributed to the –CH_2_ stretching of paraffin and this was increased in intensity with increasing paraffin contents. The positive cross peak at (2930, 3500) cm^−1^ indicated that the hydroxyl groups intensity increased in system (II) with increasing PS contents confirming the previous conclusion obtained by analysing the hydroxyl related bands. On the other hand, the band at 2930 cm^−1^ in system (II) was attributed to the –CH_2_ stretching of PS chains and this was increased in intensity with increasing PS contents. The negative cross peak at (3500, 2930) cm^−1^ indicated that the hydroxyl groups intensity decreased in system (I) with increasing paraffin contents. It should be mentioned that the previous conclusions were very difficult to reach by solely analyzing the one dimensional FTIR spectra.

Similar conclusions were obtained by analyzing the region from 1550 to 1750 cm^−1^, where four negative cross peaks at (1616, 1616), (1637, 1616), (1637, 1637), and (1616, 1637) cm^−1^ were observed. This confirmed that surface hydroxyl groups were changed in the opposite direction in both systems, and this had a role in the contact angle values.

**Figure 9:**
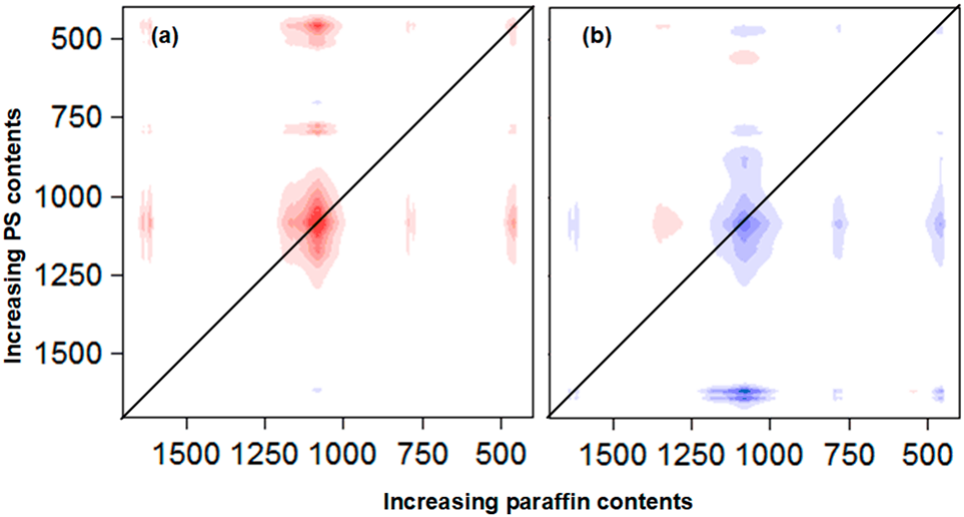
(**a**) Synchronous and (**b**) asynchronous hybrid 2D correlation spectra in the range from 1700 to 400 cm^−1^ constructed from the intensity of FTIR bands of sand coated paraffin/PS with varying amounts of PS or paraffin.

The important features in the region from 1700 to 400 cm^−1^ were discussed, see Fig. 9a and b. All bands related to quartz (1085, 798, 780 and 462 cm^−1^) correlated to each other with positive signs in both systems and were decreased in intensity. These illustrated the reduction in crystallinity of sand. However, it was observed that both bands at 1085 and 462 cm^−1^ changed more along the z axe (increasing PS contents) while other bands at 780 and 798 cm^−1^ changed more along the x axe (increasing paraffin contents). In addition, in the region from 4000 to 400 cm^−1^, there were two interesting cross-peaks; one of which was positive that correlated the bands at (3500, 1085) cm^−1^, and the other was negative that correlated the band at (1085, 3500) cm^−1^. These indicated that the hydroxyl groups were increased in system (II) but decreased in system (I), while the band at 1085 cm^−1^ was decreased in both system (I) and (II).

Yan et al.^39^ showed that the band at 1085 cm^−1^ should be assigned to SiO_4_ tetrahedron base breathing stretching vibration, while the band at approximately 462 cm^−1^ should be attributed to the O–Si–O bending mode of the SiO_4_ tetrahedron. The distortion of the tetrahedral sheet leads to the reduction of intensity and eventually disappearance of these bands.

In fact, three types of Si–O–Si bridging have been reported^40,41^. The first occurs at 1170 cm^−1^ and connects the two inverse SiO_4_ tetrahedrons, the second occurs at 1120 cm^−1^ and connects two SiO_4_ tetrahedrons in the two pyroxene chains, and the third occurs at 1033 cm^−1^ and connects the two SiO_4_ tetrahedrons in a single pyroxene chain. It was concluded that PS had much more effects in distorting the tetrahedral sheets, and subsequently reducing the crystallinity of sand. On the other hand, the two bands at 798 and 780 cm^−1^ have been previously described as the Si–O–Si symmetric stretching vibration of quartz. They are overlapped with the third type of Si–O-Si bridging^40^. In addition, the application of the sequential order rule by observing the signs of correlation peaks in both synchronous (Fig. 9a) and asynchronous (Fig. 9b) spectra led to the following order of peak change: 1085 cm^−1^ (system (II)) → 780 cm^−1^ (system (II)) → 1085 cm^−1^ (system (I)) → 780 cm^−1^ (system (I)). It was concluded that PS affected both the first and second type of Si–O–Si bridging that occurred between chains.

#### Hetero 2D-FTIR-XRD-COS

**Figure 10:**
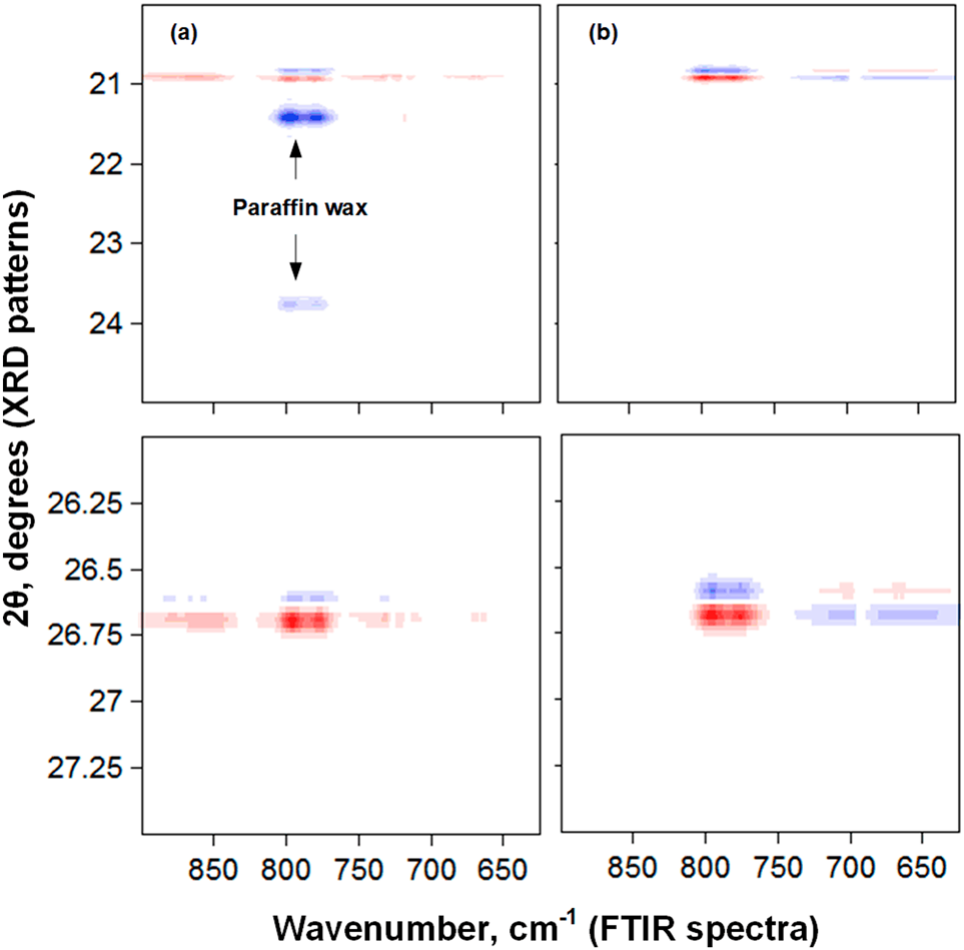
The synchronous hetero correlation FTIR-XRD spectra of (**a**) system (I) and (**b**) system (II) in the FTIR region from 650 to 900 cm^−1^ and in the XRD range from 20° to 28°.

The aims of using hetero correlation were to confirm the assignments of XRD paraffin wax peaks and to provide other evidence regarding the presence of spiliting of peaks. The assignments of the new peaks would be more easier if their origin was determined, i.e. XRD is more sensitive to change in morphology/crystallinity than FTIR. Figure 10a shows the hetero 2D-COS-FTIR-XRD synchronous spectrum of system (I). The presence of negative correlation peaks that correlated the two FTIR bands at 780 and 798 cm^−1^ with the XRD patterns at 21.4° and 23.8° indicated that both peaks at 21.4° and 23.8° have to be attributed to paraffin, which was consistent with literature^38^. The disappearance of these correlation peaks in Fig. 10b, which shows the hetero 2D-COS-FTIR-XRD synchronous spectrum of system (II), confirmed the previous assignments.

Moreover, there was splitting along the XRD axe, such that each XRD pattern split into two components. This splitting behaviour was more pronounced in system (II) and was characteristic solely for sand patterns. For example, the pattern at 20.9° produced the pattern at 20.8°, and the pattern at 26.7° produced the pattern at 26.6°. The intensity of the new peaks was increased while that of original peaks was decreased, indicating the transformation of morphology to less ordered or more distorted form.

## Conclusions

The present work provide a way to produce hydrophobic sand and at the same time reduce the plastic waste. The following four major points were concluded:

I. Natural sand can be converted into hydrophobized materials using different coating systems: paraffin wax, PS, PE and a mixture of them. The contact angles increased in the following trend: PS < PE < PE + paraffin < paraffin < PS + paraffin.
II. The hybrid 2D-FTIR-COS analysis revealed that PS affected both the first and second type of Si–O-Si bridging mode causing distortion of the tetrahedral sheets in sand structure.
III. The hetero 2D-FTIR-XRD analysis indicated that the addition of PS caused each XRD patterns to split into two components. Also, it was noticed that PS had reduced both sand crystallinity and coating paraffin thickness.
IV. The hydrophobicity of coated sand was suggested to proceed as follow: The non polar paraffin forced the hydroxyl groups to reoriented inside the sand structure near to the polar Si–O–Si bonds and crystalline water. The presence of a small amount of PS strengthened this mechanism. it is emphesized that PS acted as a compatibilizer and formed a bridge between hydrophilic sand and hydrophobic parraffin wax.

## Data Availability

The datasets generated during and/or analysed during the current study are available from the corresponding author on reasonable request.
